# Relation between health literacy, self-care and adherence to treatment with oral anticoagulants in adults: a narrative systematic review

**DOI:** 10.1186/s12889-018-6070-9

**Published:** 2018-10-04

**Authors:** Ana Cristina Cabellos-García, Antonio Martínez-Sabater, Enrique Castro-Sánchez, Mari Kangasniemi, Raul Juárez-Vela, Vicente Gea-Caballero

**Affiliations:** 1 Unidad de cuidados intensivos, Hospital Universitario y politécnico La Fe, Valencia, Spain; 2 Nursing Department, University of Valencia, Valencia, Spain; 3 NIHR Health Protection Research Unit in Healthcare Associated Infection and Antimicrobial Resistance at Imperial College London, Du Cane Road, W12 0NN, London, UK; 4 Department of Nursing Science, Faculty of Health Sciences, University of Eastern Finland, Kuopio, Finland; 5 Universidad San Jorge de Zaragoza, Villanueva de Gállego, Zaragoza, Spain; 6 Escuela de Enfermería La Fe, centro adscrito Universidad de Valencia, Valencia, Spain; 7 Instituto de Investigación La Fe. Grupo de investigación GREIACC, Valencia, Spain

**Keywords:** Health literacy, Oral coagulation therapy, Self-management, Self-care, Adherence, Systematic review

## Abstract

**Background:**

Oral anticoagulants (OAC) are widely used in patients with cardiovascular diseases. However, for optimal OAC self-care patients must have skills, among which health literacy (HL) is highlighted. We aimed to describe the relation between HL and self-care in cardiovascular patients on OAC treatment.

**Methods:**

Electronic searches were carried out in the PubMed, Scopus, Embase, CINAHL, Web of Science, Cochrane Library, SciELO, IME-Biomedicina, CUIDEN Plus and LILACS databases, limited to Spanish and English language and between January 2000–December 2016. Papers reported on adults older than 18 years, taking OAC by themselves for at least three months. PRISMA guidelines were used for paper selection.

**Results:**

We identified 142 articles and finally included 10; almost all of them about warfarin. Our results suggest that in patients taking OAC treatments there is a positive relationship between HL and the level of knowledge. In addition, a small percentage of participants on the selected papers recognized the side effects and complications associated with OAC treatment. Lower HL level was associated with greater knowledge deficits and less adherence to treatment.

**Conclusion:**

There is a paucity of research evaluating the effect of HL on diverse aspects of OAC treatments. There is a need to expand the evidence base regarding appropriate HL screening tools, determinants of adequate knowledge and optimal behaviours related to OAC self-management.

**Electronic supplementary material:**

The online version of this article (10.1186/s12889-018-6070-9) contains supplementary material, which is available to authorized users.

## Background

According to the World Health Organization (WHO), cardiovascular diseases are the leading global cause of death. Among the cardiovascular health problems, atrial fibrillation (AF) is the most frequent arrhythmia [1, 2] being associated with high mortality and morbidity and the chief cause of embolic events. Estimates suggest that the prevalence of AF in Europe will increase by 60% between 2010 and 2040, [3, 4] which may explain the increased use in recent years of highly effective therapies such as oral anticoagulation (OAC) therapies [5, 6].

OACs are used to maintain adequate coagulation levels and thus prevent thrombotic episodes. These drugs present particular characteristics (daily dose with great variability, narrow therapeutic range, complex pharmacokinetic and pharmacodynamic profile and possibility of both thrombotic and haemorrhagic complications) that make necessary periodic blood tests and careful clinical control. The International Normalized Ratio (INR) blood test is used to discern the effectiveness and safety of the treatment, with results between 2 and 3 considered adequate. When 60% of INR measurements in a patient are within such range (denominated Time in Therapeutic Range, TTR), the underlying cardiac problem is considered to be well controlled if the measurement is performed by the direct method (percentage of controls in range) during a valuation period of at least 6 months. If measurements are calculated by the Ronsendaal method, then guidelines recommend TTR > 70% [1, 5–8]. Adequate health knowledge and patients’ self-care are therefore vital to maintain an optimal treatment concordance due to the complexity of the condition and drug characteristics.

Health literacy (HL) has been identified as a crucial determinant of public and individual health as well as self-care [9, 10]. Conceptually, HL is dynamic and refers to the knowledge, motivation, and competencies to act, understand, evaluate and apply health information to care-related decisions [11–13]. Currently, different tools are used to measure HL levels encompassing communication skills, information search capability, and previous experience of health care [14–16]. In recent years, the emphasis has been placed on the adaptation of health and social care systems to the HL of the population, to facilitate and optimize human and material resources and improve clinical and health outcomes.

Some preliminary studies exploring the effect of HL on awareness about AF and medication concordance suggest that patients with low HL were less likely to take treatment as recommended, therefore increasing the risk of complications and disease-related mortality [17]. These results match well those reported in other chronic diseases where people with low HL experienced worse health status and outcomes, [18, 19] suboptimal management of treatment with direct consequences such as medication errors, [20] higher risk of hospital admission, [21] increased social costs, [22, 23] less use of preventive services and in essence higher mortality. [24–26] The association between low HL and decreased knowledge regarding factors of disease prevention, medicines use, the importance of dose adjustment and adherence to treatment has also been described [27–29].

The aim of this systematic review was to describe the influence of HL levels in the self-care of cardiovascular pathologies managed with OAC treatment. The research questions were:

1. In patients taking OAC treatments, what is the relationship between HL levels and self-management/self-care, adverse effects and complications?
2. What instruments have been used to determine levels of health literacy in patients taking OAC treatments?
3. What has been the impact of health interventions tailored to the level of HL in the adherence to OAC treatments, as evidenced by changes in INR results?

## Methods

We conducted a systematic review following PRISMA guidelines [30]. In the first stage, we built a search strategy with the following PICO approach (Table 1).

**Table 1:** Overall structure of the systematic review

P (patients)	Adults patients on oral coagulation therapy
I (intervention)	Measures to assess and improve health literacy
C (comparison)	Inappropriate due to there are no concepts to compare
O (outcome)	Self-care and medication adherence

### Literature searches

**Fig. 1:**
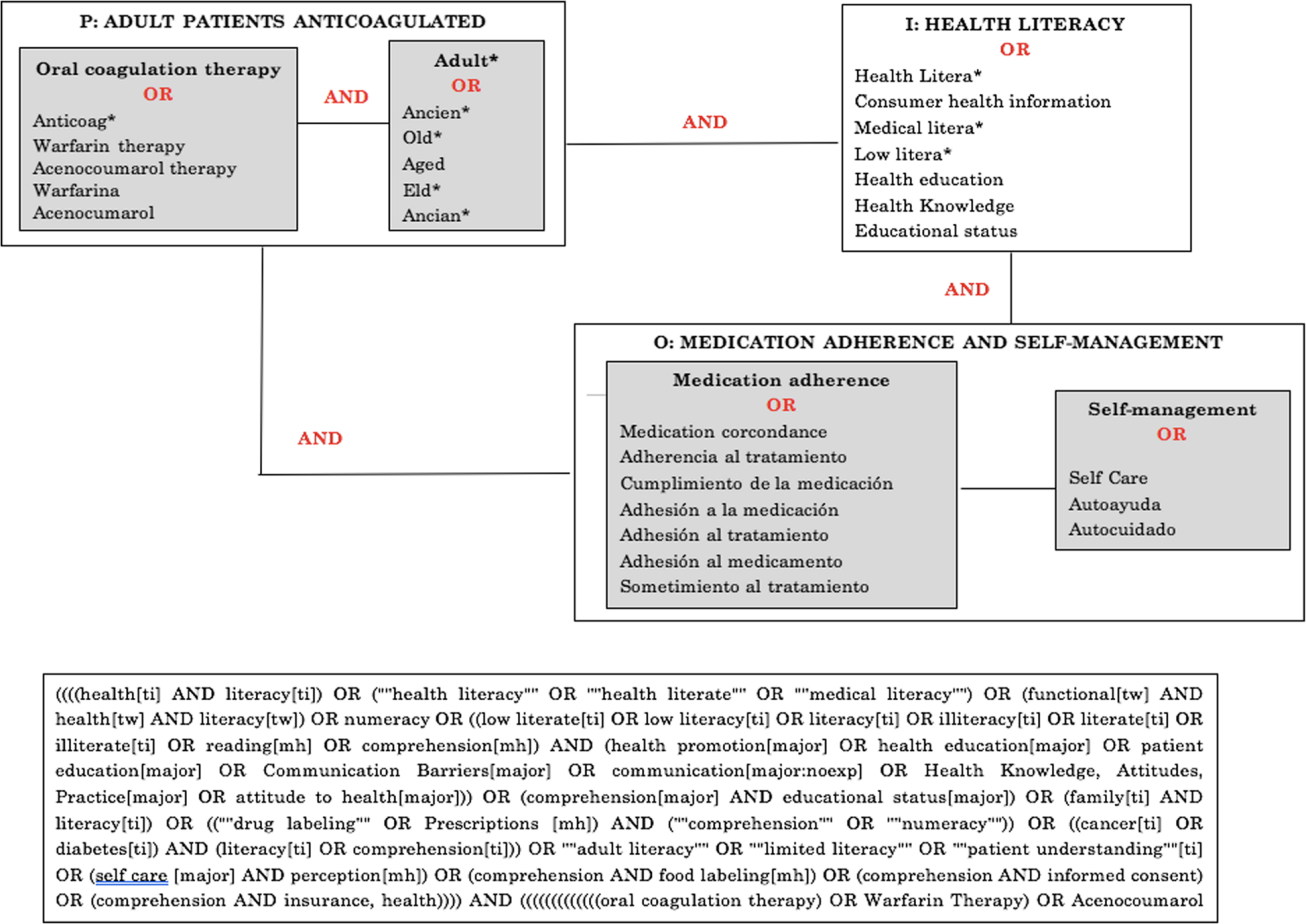
Systematic review search strategy and keywords

We searched the electronic databases PubMed, Scopus, Embase, CINAHL, Web of Science, Cochrane Library (all these in English), and SciELO, IME-Biomedicina, CUIDEN Plus and LILACS (in Spanish). Search terms were identified used the DeCS, MeSH and Tesauro PsycINFO tools, combining the terms ‘adult patients’, ‘oral coagulation therapy’, ‘health literacy’, ‘self-care’, ‘self-management’ and ‘medication adherence’, and appropriate synonyms in English and Spanish. We combined the search terms with Boolean operators “OR” and “AND” (Fig. 1).

We included papers which focused on i) patients older than 18 years, ii) on oral coagulation therapy for at least three months, and where iii) patients were taking medication by themselves. Additionally, papers had to report on studies conducted in iv) primary healthcare settings using v) qualitative or quantitative methods, including economic studies. We excluded papers that focused on i) patients with neurocognitive impairments, dementia or mental health disorders and ii) inpatients or patients admitted to hospitals or similar settings.

As limits to the search, the papers had to be published in Spanish or English between January 2000 and December 2016 in a peer-reviewed scientific journal. In addition, an abstract had to be available.

### Data evaluation

After completion of paper selection, we evaluated the quality of manuscripts with ICROMS tool (Integrated Quality Criteria for the Review of Multiple Study designs) for assessing Risk of Bias [31], which has been used previously in various articles [32, 33]. Data were selected and analysed by two authors working independently. In case of disagreement, a third author was consulted.

### Data analysis

As a first stage of analysis, the selected papers were read several times to get a clear picture and understanding. After that, information within the papers was tabulated in a standardised form according to authorship, year, country, aims, methodology, and population and sample characteristics. In addition, we included information about the instrument used to assess HL, OAC treatment, interventions/activities, risk of bias and results (Tables 2 and 3).

**Table 2:** Summary of the selected studies/papers

Author, year. Aim	Methods	Instrument for measuring HL	OAC medication	Risk of Bias (according to ICROMS tool)
Diug, Evans et al. 2011 Evaluate impact of psychosocial factors (social isolation, HL, cognitive abilities) in OAC treatment.	^a^Case-control study. Control: Patients with INR in therapeutic range. Cases: previously stabilized patients who registered INR ≥ 6 ^a^Sample: 486 patients (157 cases and 329 controls) ^a^Structured interview of 1 h to identify risk factors. Interview after 30 days. ^a^Data collection: - Medical history (INR) - Socio-demographic data - Comorbidities, cognitive function…	^a^S-TOFHLA - Limited HL (0–22 points) - Adequate HL (23–36 points)	^a^Warfarin	^a^Possible Patient memory bias ^a^Neither interviewers nor patients blinded to obtain cases ICROMS Score: 24 S1:2; S2:2; S3: 5; S4:2; S5:2; S6:2; S7: 9
Dolor, Ruybalid et al. 2010 Evaluate if home self-control test (PST) improves the quality of anticoagulation and decreases complications	^a^Randomized Controlled Trial ^a^Sample: 2922 patients. ^a^Training to handle PST ^a^Data collection: - Medical history (INR) - SPMSQ (Assesses cognitive status) - ABILHAND (Measure dexterity) - DASS (Duke anticoagulation satisfaction scale). Quality of life	^a^REALM Scale	^a^Warfarin	^a^Only Veteran patients with AF or valve replacement (selection bias) ICROMS Score: 28 S1:2; S2:4; S3:6; S4:6; S5:1; S6:2; S7:7
Estrada, Martin-Hryniewicz et al. 2004 Determine prevalence of low HL in OAC patients and to assess if there is association with anticoagulation control.	^a^Prospective cohort study ^a^Sample: 143 patients. ^a^Data collection: - Socio-demographic data - Medical history (dose in mg/week, number of dose changes, number of visits without going, indication of anticoagulation) - INR: monitoring for 3 months. - Time Therapeutic Range (TTR) ^a^Variability of INR was measured by Sigma (INR number, time from the last INR and INR suitable for that patient)	^a^REALM Scale	^a^Warfarin	^a^REALM scale only values reading level, non-comprehension (functional HL) ^a^Only English-speaking patients were assessed. ^a^Complications are not measured. ICROMS Score: 23 S.1:2; S2:2; S3:5; S4:2; S5:1; S6:2; S7:9
Fang, Machtinger et al. 2006 Assess the association between HL and warfarin knowledge, adherence and control.	^a^Observational, descriptive study ^a^Sample: 179 patients. ^a^Data collection: - INR - S-CASI (Assesses cognitive abilities) - 4 questions regarding anticoagulation and safe use of warfarin ^a^INR control every 4–6 weeks. ^a^Adherence to treatment: last time they forgot to take warfarin, if they forgot 1 dose in the last 2 weeks or in the last 2 days.	^a^S-TOFHLA Scale. - Limited HL (0–22 points) - Adequate HL (23–36 points)	^a^Warfarin	^a^Only includes patients who speak English or Spanish ^a^Self-made questionnaire concerning knowledge of anticoagulation ^a^Self-report adherence measure (recall bias) ^a^Complications are not measured. ICROMS Score: ^a^
Fang, Panguluri et al. 2009 To assess the relationship between HL and patients with stroke with warfarin to assess perceived information and improve communication.	^a^Observational, descriptive study ^a^Sample: 183 patients. ^a^Data collection: - Medical history - Socio-demographic data - S-CASI (cognitive abilities) - Questionnaire of two open questions: why do you take warfarin and what is a stroke? Classification answers: concordant /discordant	^a^S-TOFHLA Scale. - Inadequate HL (0–16) - marginal HL [17–22] - Adequate HL [23–36]	^a^Warfarin (For at least 3 months)	^a^Only 2 open questions, insufficient to explore subjects’ perception ^a^Relationship between HL and open-ended questions not validated ^a^Sample obtained in a single center (selection bias) ^a^Only patients with stroke ICROMS Score: -^a^
Oramasionwu, Bailey et al. 2014 Assess the relationship between HL and anticoagulation control (TTR)	^a^Observational, descriptive study ^a^Sample: 198 patients. ^a^Data collection: - Medical history - INR / TTR - Socio-demographic data (annual income) - Questionnaire to evaluate understanding of anticoagulant treatment (Fang et al. 2006)	^a^S-TOFHLA (36 items of reading comprehension and 4 items of multiple questions of arithmetic). 100 points. - Limited HL: 0–90 -Adequate HL: 91–100	^a^Warfarin	^a^Not assess the appearance of complications. * Not incorporate factors that could modify INR (diet, other medicines) *^a^Only English-speaking patients were included ICROMS Score: -^a^
Schillinger, Machtinger et al. 2006 To relate verbal-visual communication with medication management	^a^Observational, descriptive study ^a^Sample: 220 patients. ^a^Data collection: - Medical history (INR) - S-CASI (Assesses cognitive abilities) - Number of days they forgot to take warfarin week prior to study	^a^S-TOFHLA Scale - Inadequate HL (0–16) - marginal HL [17–22] - Adequate HL [23–36]	^a^Warfarin	^a^Reduced sample size * Method used to determine agreement of treatment (does not determine if visual agreement greater than verbal) ICROMS Score: ^a^
Schillinger, Wang et al. 2006 To examine if there is a mismatch between the anticoagulant treatment that the patient takes and the standard. Assess adherence	^a^Observational, descriptive study ^a^Sample: 220 patients. ^a^Data collection: - Medical history (INR) ^a^-Adherence (n° of times forgot to take the treatment in 30 days) ^a^S-CASI (cognitive abilities) ^a^Agreement of treatment: evaluated concordance of the weekly mg.	^a^S-TOFHLA (English and Spanish) - Inadequate HL (0–16) - marginal HL [17–22] - Adequate HL [23–36]	^a^Warfarin	^a^A single clinic is included. ^a^Measurement of adherence subject to recall bias and social acceptability bias. ICROMS Score: ^a^
Wilson, Racine et al. 2003 To investigate the level of HL and to evaluate the readability and cultural sensitivity of the information administered in an anticoagulation clinic	^a^Descriptive, correlational study ^a^Sample: 65 patients. ^a^Designed an easy-to-read ^a^ducational/ informative material. ^a^Once a week patients were interviewed about diet, medicine control + 15 min. of education for health. ^a^Data collection: - Medical history (INR) - Socio-demographic data (annual income) - Self-made warfarin knowledge questionnaire (20 items) - SMOG formula (to assess the reading difficulty level of the guide)	^a^REALM Scale	^a^Warfarin	^a^Self-made questionnaire concerning knowledge of warfarin ^a^Reduced sample size ^a^Does not relate INR with HL. ^a^Complications are not measured. ICROMS Score: 19 S1:2; S2:2; S3:4; S4:2; S5:0 S6:2; S7: 7
Wilson, Templin et al. 2015 To evaluate the psychometric properties of the KIP-C20 test	^a^Descriptive, correlational study ^a^Sample: 192 patients. ^a^Creation KIP-C14 test. ^a^Data collection: - Socio-demographic data (annual income) - KIP-C20 test: Knowledge Information Profile-Coumadin. 20 items with response T/F. 2 weeks the KIP-C20 test was administered again. - Animal Naming Test (ANT): To assess cognitive ability	^a^REALM Scale	^a^Coumadin	^a^Use sample from a single center. (Similar economic level of patients) ^a^No complications or adherence to treatment were measured ^a^Limited to coumadin medicine ICROMS Score: 21 S1:2; S2:2; S3:4; S4:2; S5:0 S6: 2; S7:9

^a^The ICROMS tool does not include the evaluation of observational, descriptive studies

**Table 3:** Main results of the selected studies/papers

Author(s), year, country	Main results
Diug, Evans et al. 2011, Australia	• Inadequate HL, cognitive impairment and depression were associated with increased risk of bleeding (HL stronger relationship). • Inadequate HL in 68% cases and 39% controls. • Group of cases worse adherence and less use of dispensers.
Dolor, Ruybalid et al., 2010, USA	• 88.4% successfully completed training • Failure to perform PST NOT related to HL
Estrada, Martin-Hryniewicz et al., 2004, USA	• 47.6% have adequate HL and 11.2% have inadequate HL. • Positive correlation between low HL and greater variability in INR. • HL level not associated with time remaining in range.
Fang, Machtinger et al., 2006, USA	• 60.9% have limited HL • Median S-TOFHLA: 17 • Limited HL was associated with knowledge deficit of its pathology (AF) • Limited HL was not associated with INR in range or adherence.
Fang, Panguluri et al., 2009, USA	• S-CASI < 17 was associated with discordant responses in stroke and warfarin therapy. • Average score of S-TOFHLA:17 (marginal HL) • Inadequate HL was associated with discordant responses
Oramasionwu, Bailey et al., 2014, USA	• Patients with limited HL have an older age, lower level of education, and lower annual income. • 51% have limited HL. • Limited HL was associated with worse control (TTR < 50%) in adults> 65 years.
Schillinger, Machtinger et al., 2006, USA	• 48% inadequate HL and 13% marginal HL • 39% adequate HL • 56% INR in range for 90 days • Minor visual and verbal agreement in patients with insufficient HL
Schillinger, Wang et al., 2006, USA	• 48% inadequate HL, 13% marginal HL and 39% adequate HL. • 70% maintained adherence but 50% presented discordant regimes • 43.8% of INR was out of range.
Wilson, Racine et al., 2003, USA	• 90% knew that warfarin was anticoagulant, only 50% Knew side effects • Significant relationship between HL and knowledge level • HL greater in women
Wilson, Templin et al., 2015, USA	• 52.9% score REALM between 45 and 60. • 72.8% responded correctly to the KIP-C20 • Correlation between KIP-C14 and REALM was 9%. • Important gap in HL level.

## Results

### Search outcome and selection

**Fig. 2:**
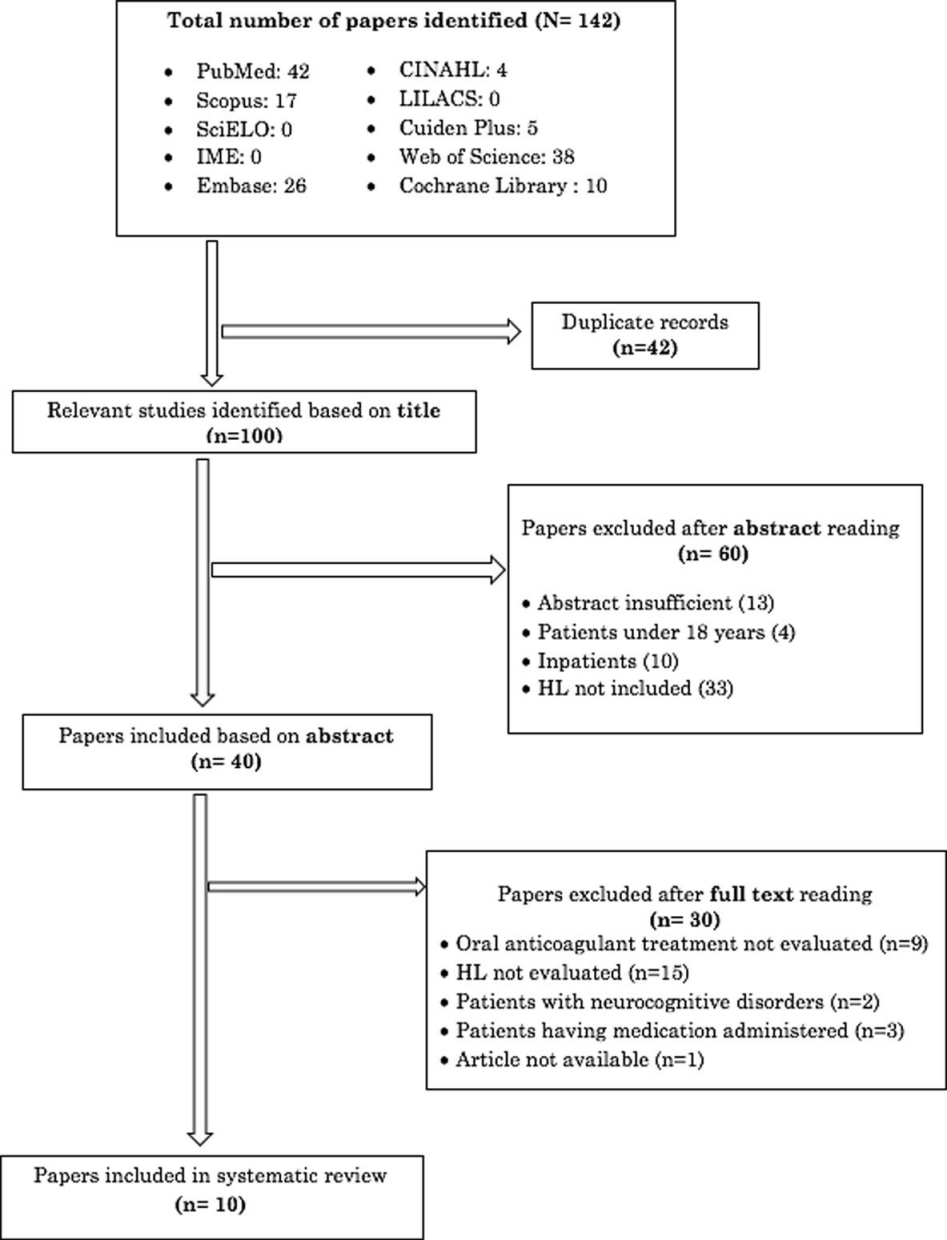
Systematic review results chart

Figure 2 presents the study flow. Based on the electronic searches, we identified 142 original papers. Following removal of duplicates, 100 papers were title screened, 40 papers were abstract screened, and finally 10 papers were full-text screened.

### Methodological approaches of studies selected

All 10 original papers selected were quantitative. The most frequently used study design was descriptive (*n* = 7), including cross-sectional designs usually adjusted for age, sex and level of studies and with little inclusion of inferential methods. Three studies conducted longitudinal analyses, one of which was a randomized trial, [34] one cohort study [35] and one case-control study [36].

The variables collected in the studies were mainly sociodemographic, including the level of income in four articles [36–39]; others also collected data about medical history, including INR or TTR, although two articles did not have this marker [39, 40]. Different tools were used to assess cognitive ability, including the Cognitive Abilities Screening Instrument-Short Form (S-CASI), Short Portable Mental Status Questionnaire (SPMSQ), Animal Naming Test (ANT) and the ABILHAND (to measure of manual ability for adults) [34, 39–43]. In terms of geographical location, nine studies were conducted in United States (US), [34, 35, 37–43] and one was originated from Australia [36]. No papers published in Europe were found. The most recent articles were published between 2014 and 2015 [37, 40].

### Methodological quality

The 10 studies identified presented an acceptable methodological quality, following evaluation with the PRISMA guidelines and ICROMS tool to assess the Risk of Bias (Table 2). Selection and recall bias were the most commonly identified biases, present in two papers [34, 39] and [41, 43] respectively. One other paper presented social acceptability bias [43]. In addition, two articles included a rather small sample size [38, 42].

### Health literacy measurement tools

All manuscripts included in the review used validated tools to measure HL. However, the tools were validated only in English-speaking or Spanish-speaking patients residing in the US. Of note, there were no tools validated in other Spanish-speaking populations. The most frequently validated tool used to measure HL was the short version of the Test of Functional Health Literacy in Adults (S-TOFHLA), present in six articles [36, 37, 39, 41–43] and the Rapid Estimate of Adult Literacy in Medicine (REALM) scale used in four articles [34, 35, 38, 40].

### Epidemiology of health literacy

In seven studies about 50–60% of participants had limited or inadequate HL, [36, 37, 39–43] studies used S-TOFHLA, whilst one employed the REALM tool. [40] Three studies (using REALM) obtained a prevalence of limited HL of 12–15% and adequate HL of around 70–80% [34, 35, 38].

In terms of the relationship between HL and the age of the participants, five studies [35, 37–39, 41] reported an inverse relation between these variables. Further, women had lower HL levels in all except one study [38]. Regarding the association between HL and level of education, all studies demonstrated a direct relationship. One study qualified that participants with limited HL had lower levels of education, less employment, and lower annual income [37]. All selected studies directly related HL levels to educational or reading levels. In four studies the participants’ annual income was included, [36–38, 40] but only one paper directly related it to the level of HL [37].

### Knowledge and sources of information

Focusing on the health-related knowledge of participants and the different mechanisms used by them to obtain information, approximately 50% of study participants received both written and verbal information from health professionals. The impact of such information however was less evident; one of the studies reported that 90% of patients knew which of their medicines was an oral anticoagulant, yet 70% were unaware of the need to monitor potential food interactions and a further 18% did not understand the side-effects [38]. Despite these gaps, 40% of participants felt their level of knowledge about treatment was good or moderate, which could lead to inequalities or discrepancies between their level of care and the interventions or recommendations implemented by healthcare professionals.

### Relationship between HL level, self-management of OAC and adverse effects

Regarding the type of oral anticoagulant treatment reported, the most commonly used medication was warfarin (nine studies), [34–39, 41–43] with Coumadin used in one study [40].

### Relationship between HL level and self-management

When analysing the relationship between levels of HL and self-management of OAC treatments, the studies reported a relation between lower levels of HL, deficits of knowledge and increased risk of health problems such as bleeding and non-specific side effects or suboptimal treatment adherence. [35–37, 41, 42] Eight studies included INR measurements, with 30–50% of participants within adequate therapeutic range. [34–38, 41–43]; however, four studies [34, 38, 42, 43] did not link INR results to HL. Four studies [35–37, 41] established a relationship between the HL of participants and OAC treatment with contradictory results.

### Relationship between HL level and INR

In the study by Fang et al. [41] with a sample of 179 patients in which INR control was performed every 4–6 weeks, a limited level of HL was not associated with INR within adequate range. The study by Oramasionwu et al. [37], on the other hand, with a sample of 198 adults older than 65 years but without clear information about the frequency of INR control suggested that limited HL was associated with poorer therapeutic control (TTR < 50%). Finally, Estrada et al. [35] found that INR variability was 32% higher in patients at the lowest literacy level as compared with patients at the highest literacy level, but HL level not associated with time remaining in range, in a study of 143 patients where INR was monitored quarterly. In Australia, a case-control study reported that poor health literacy was the strongest relationship related to a higher INR and therefore higher bleeding risk. [36]

### Relationship between HL level, adherence and adverse effects

With regards to adherence to the therapeutic regime, four studies evaluated this aspect obtaining disparate results. Two papers identified an association between HL and adherence to anticoagulant treatment, with lower HL linked to suboptimal adherence [36, 42].

Adverse effects and associated complications were examined by other two studies [36, 38]. In terms of participants’ knowledge of possible complications or side effects, these studies were in agreement reporting a low percentage of participants who recognized adverse events and had difficulty adequately controlling INR. In the study by Wilson et al. [38], between 30 and 50% of participants were unaware of crucial side effects, whilst the study by Diug et al. [36] identified an association between inadequate levels of HL and increased risk of haemorrhage. This study also explored the impact of psychosocial factors and depression on self-management of OAC treatment. In the study, about 40% of participants had depression and it was associated with poorer control of treatment, poor adherence, inadequate HL, lower satisfaction and greater side effects, mainly increased risk of bleeding.

## Discussion

Our review centred on the relation between HL, self-management and OAC treatment outcomes. We identified a limited number of studies on this topic, generally conducted in the US and focused on a rather small sample size (around 150 patients). There appeared to be large variability in the relationship between HL and different aspects of self-management of ACO treatment. The limited evidence available suggests a positive relationship between HL and the level of knowledge presented by patients on ACO treatment, in line with other pathologies where these aspects have been studied.

The results obtained in our review present some limitations, mainly due to the cross-sectional design of most studies in which HL measurement tools were used. As the description of results with respect to HL levels was often not detailed, comparison across studies may be inaccurate. In addition, HL was most frequently associated with knowledge only, yet the concept includes many other domains. Finally, the included studies lack generalizability to other settings and healthcare systems as most of them were conducted in the US.

HL was evaluated using mainly two validated tools (S-TOFHLA and REALM). A mix of tools were in place to examine different aspects about OAC treatment, including the Duke Anticoagulation Satisfaction Scale, [34] the questionnaire to evaluate understanding of anticoagulant treatment by Fang et al. [37], the KIP-C20 [40] and different other ad hoc questionnaires. In general, the methodologies and designs of the publications concur with previous studies evaluating HL in other health problems [44]. We therefore encourage the development and reporting of research where the relation and impact of factors such as self-care, treatment duration, disease stage, disease aetiology and HL level are considered. In addition, we highlight the lack of studies where interventions aimed at mitigating the impact of low HL on clinical and health outcomes are described [45, 46].

Despite the positive association between HL and the level of knowledge that all studies reflected, it is important to note that whilst different components and skills that inform or determine HL were evaluated in some of the studies discarded [47–51] HL was not specifically measured, which can make it difficult to make comparisons and lead to confusion. Paradigmatically, one of those studies [51] used two specific HL questions and assessed the readability of materials. However, it is increasingly acknowledged that HL is a much broader concept than readability and encompasses other social and critical elements [52]. As another difficulty, the different studies included a variety of HL strata, mostly using categories such as “inadequate”, “marginal” or “adequate” but also “high” or “low”, with several cut-off points stratifying HL levels. Additionally, the studies focused primarily on the differences between groups with higher or lower HL, rather than elaborating on the differences between the different groups.

Additionally, there seems to be a growing interest in developing validated, pathology-specific, HL measurement instruments, as seen for example in diabetes, rheumatism, colon cancer, hypertension, and Human Immunodeficiency Virus (HIV) [52]. Whether such efforts are warranted would demand theoretical underpinnings demonstrating that HL skills associated with those health problems or others are different to the domains included in general HL screening tools. For the moment, and focusing on OAC treatments, there is no validated tool for the evaluation of health literacy in cardiac pathologies such as AF.

The review reveals a widespread absence of evaluation between HL and subsequent complications or increased hospital admissions. When such association was included, it was found to be weak. Such findings mirror the conclusions of a systematic review assessing the relationship between HL and diabetes outcomes, [53] with limited evidence linking HL and serious clinical events. In contrast, another systematic review [52] postulated that low HL levels were associated with increased use of health services, including hospitalizations and use of emergency facilities.

Our review identified some inconsistencies between HL levels and adherence to treatment. In the four studies that quantified such association, only two demonstrated a positive association. Some factors that could explain such disparity may include the different adherence assessment methods used, including self-reported tools which may lead to recall bias. Regarding INR control, the two studies measuring this relationship diverged in their conclusions, with one experience linking limited HL to worse TTR control [37] and another not identifying any association between health literacy and TTR. [54] Finally, a slightly different approach was reported by Tang et al. [55] who positively correlated the level of patient knowledge and the frequency of INR measurements within the appropriate range. Interestingly, studies in other clinical areas have arrived at similar range of results. For example, whilst HL was associated with better adherence to glaucoma treatment [56] and glycaemic control, [57] this was not the case in experiences focused on HIV therapy or oral contraception [58].

## Conclusion

Even considering the narrow scientific evidence and the limitations of the studies found, it seems appropriate to suggest that improving HL levels among patients taking OAC treatment would lead to increased self-management and therefore facilitate optimal use of health services. To achieve such goal, healthcare organisations should evaluate their resources and clinical pathways to ensure that patients with low HL can be supported and any associated inequalities in outcomes are addressed. In parallel, it may also be essential to tackle the impact of main determinants of HL such as education and socioeconomic status to ensure that patients are able to mitigate risk factors contributing to pathologies which require OAC treatments.

## Additional file

Additional file 1: PRISMA checklist. (DOC 63 kb)

## Abbreviations

AF: Atrial fibrillation
ANT: Animal naming test
DASS: Duke anticoagulation satisfaction scale
DeCS: Descriptores en Ciencias de la Salud
ESC: European Society of Cardiology
HIV: Human immunodeficiency Virus
HL: Health literacy
ICROMS: Integrated quality Criteria for the Review of Multiple Study designs
INR: International normalized Ratio
KIP-C20: Knowledge Information Profile–Coumadin
MeSH: Medical subject Headings
OAC: Oral anticoagulation
PRISMA: Preferred reporting Items for Systematic Reviews and Meta-Analyses
PST: Patient self-testing
REALM: Rapid estimate of Adult Literacy in Medicine
S-CASI: Cognitive abilities Screening Instrument-Short Form
SMOG: Simple measure of Gobbledygook
SPMSQ: Short portable Mental Status Questionnaire
S-TOFHLA: Test of Functional Health Literacy in Adults
TTR: Time in therapeutic Range
US: United States
WHO: World Health Organization
